# Integrated Care in the Community: The Case of the *Programa Maior Cuidado* (Older Adult Care Programme) in Belo Horizonte-Minas Gerais, BRA

**DOI:** 10.5334/ijic.5619

**Published:** 2021-06-21

**Authors:** Janaína de Souza Aredes, Jenny Billings, Karla Cristina Giacomin, Peter Lloyd-Sherlock, Josélia Oliveira Araújo Firmo

**Affiliations:** 1 Centre for Studies in Public Health and Aging, René Rachou Institute, Fiocruz, Minas Gerais, BRA; 2 Centre for Health Services Studies, University of Kent, Canterbury, Kent, UK; 3 Belo Horizonte Municipal Health Secretary, Minas Gerais, BRA; 4 University of East Anglia, School of International Development, Norwich, UK

**Keywords:** integrated care, older vulnerable people, health and social integration, Brazil

## Abstract

Internationally, there is a large body of scientific evidence concerning the benefits of integrating health and social care to ensure that frail older people living in the community receive the assistance they need to maintain independence. In the Brazilian city of Belo Horizonte, located in the state of Minas Gerais, an integrated care intervention has been developed: the *Programa Maior Cuidado – Older Adult Care Programme* (PMC). This programme represents a pioneering example in Brazil of the provision of carers for highly vulnerable older people, through integrated action between public health and social service agencies. This paper draws on the first phase of a mixed method evaluation of PMC, including data from documentary sources, focus groups, empirical observation and expert workshops, to examine the processes that led to the establishment of programme. The origins of the PMC are discussed and its operational processes, with a particular emphasis on integrated activities and the roles of different actors. The paper situates PMC within comparable international experiences of integrated provision for older people and considers how it has been affected by unique context and challenging of a middle-income country.

## Introduction

Rapid population ageing represents a significant challenge for most low and middle- income countries. By 2015, 62% of people aged 70 or more were living in less developed regions, and this is projected to reach 76% in 2050 [1]. As in high-income countries, an ageing population requires policies to promote integrated health and social care, to support independent living at home and reduce unnecessary hospital admissions [2]. Numerous studies refer to the potential benefits of integrating health and social care, including more efficient use of health services and improved health outcomes for older people and their carers. These integrated interventions can take many forms, but mainly focus on collaboration between health and social assistance professionals, and with family carers [3,4,5].

Brazil currently has the 6^th^ largest population of older people in the world, with approximately 30 million people aged 60 or older – 15% of the country’s total population. By 2030, this number will surpass the number of children and adolescents and by 2050 it will reach 64 million people, 30% of Brazilian population [6]. Over the next 25 years, the share of people aged 70 or older will treble, to reach 16.4% – more than current levels in more developed regions [7]. Brazil is ageing in a context of deep economic crisis, and profound social and gender inequality, all of which present challenges to public policies. Austerity policies, similar to those in Europe, are being implemented, which may further compromise the provision of care to the most vulnerable population groups [8]. All of the above have been exacerbated by the Covid-19 pandemic, which has hit Brazil hard, with the majority of deaths occurring among people aged 60 or more [9].

As in many countries, Brazil has separate administrative systems for health services and for social care: the Unified Health System (*Sistema Único de Saúde* – SUS in Portuguese) established in 1990 and the Unified System for Social Assistance (*Sistema Único de Assistência Social* – SUAS in Portuguese) established in 2004.

There are national mechanisms to support coordination and learning between local governments and responsibility for this is mainly decentralised to the municipal level. These mechanisms focus on community-based primary health care, involving interdisciplinary Family Health Teams (*Equipe de Saúde da Família* – ESF in Portuguese) of doctors, nurses, community health agents (*Agente Comunitário de Saúde* – ACS in Portuguese), social workers, nutritionists, physiotherapist and psychologists [10]. Each team offers a comprehensive set of primary health care services to defined communities of 3000 to 4000 inhabitants. These ESFs are responsible for referral, coordinating different SUS health services and operating as a bridge between the health system and local communities [10]. The local institutional hub for this system is the Health Centre (*Centro de Saúde* – CS in Portuguese), which provides primary health care to designated geographical populations [10].

SUAS has a somewhat wider set of roles than social welfare agencies in many high-income countries, including the management of national cash benefit schemes and the provision of social services. It has a similar decentralised structure to that of the SUS, with national coordination and local hubs for community engagement. Local hubs known as Social Assistance Reference Centres (*Centro de Referência em Assistência Social* – CRAS in Portuguese) perform a similar role to SUS Health Centres (CS), offering a wide range of services for people of all ages, with a particular emphasis on protecting and strengthening relationships between family members and guaranteeing human rights [11]. Unlike CS, however, it does not operate in all parts of Brazil: its coverage is restricted to areas defined as highly vulnerable and at social risk, such as precarious urban neighbourhoods.

In Brazil, even before the Covid-19 pandemic, there was growing awareness of the need to manage pressures on the SUS in the context of population ageing [12]. Rates of health service use and many preventable hospital admissions of older people [3] are higher for older Brazilians than for the population in general [13]. Different studies report that unnecessary hospital admissions and protracted lengths of hospital stay are in part due to a lack of family support or social care in the community at home [14,15,16]. Unnecessary hospital stays expose older people to other health risks and contravene the preferences of many older people to remain in their own homes as much as possible [16,17]. To date, Brazil has made little progress to integrate health and social care services for older people at the national level. However, the decentralised structures of SUS and SUAS allow local governments some scope to develop their own policy interventions to promote an integrated approach. The most notable example of this is in the city of Belo Horizonte, which has been running an innovative intersectoral scheme, *Programa Maior Cuidado* (PMC – Older Adult Care Programme), for nearly ten years.

Implemented in 2011 as a partnership between Belo Horizonte’s municipal departments of health and social assistance, PMC represents a novel approach for managing social care for highly vulnerable older people in Brazil [18]. PMC originated from discussions held by an Intersectoral Working Group formed in 2010, composed of representatives of different agencies (Education; Health; Social Assistance; Citizenship; Culture, Sports and Leisure) and the Municipal Council of Older People’s Rights. This group aimed to develop strategies for supporting families with frail older people in situations of social vulnerability – as a consequence of weak family/social ties or limited opportunities for inclusion in the community, generating situations of risk, exclusion and social isolation [18,19].

This paper draws on the first phase of a evaluation of PMC, including data from documentary sources, focus groups and empirical observation, to examine the processes that led to the establishment of PMC. We also compare how PMC functions in theory to the actual experiences of different health and social assistance professionals involved in its day to day operation. This pays particular attention to the degree PMC has promoted enhanced service integration. It also considers the specific setting in which this integrated programme has been developed. The vast majority of literature on comparable interventions refer to high-income countries [3]. Brazil, and more specifically, the city of Belo Horizonte represent a distinct context. With a population of around 2.5 million people, Belo Horizonte is the sixth largest city in Brazil and, as elsewhere in the country, is characterised by high levels of poverty, social exclusion and inequality [20]. This is most evident in the city’s most deprived neighbourhoods, which are where PMC operates. As such, a key contribution of this study is the unique insight it provides into processes of service integration for older people in a highly challenging middle-income country setting.

## Study design

This is an exploratory study, [21] seeking to map the organisation and functioning of PMC and to reveal the nature of relationships between key stakeholders and actors in different participating agencies. It is part of a wider evaluation project, “Improving the effectiveness and efficiency of Health and social care services for vulnerable Older Brazilians – IHOB” (2018–2021) approved by the René Rachou Research Ethics Committee, Fiocruz-MG (CAAE: 96033418.9.0000.5091). The research team is multidisciplinary, including social scientists, geriatricians, a nurse, health services managers and public policy makers with interests in ageing. The main objective of the larger study is to provide evidence to support policies to reduce unnecessary stays of older people in hospitals and long-term care facilities. These include admissions for conditions that could be treated outside inpatient settings, admissions for conditions that could reasonably be prevented by adequate community-based health and social care, and delayed discharge due to a lack of home-based support [2].

**Figure 1:**
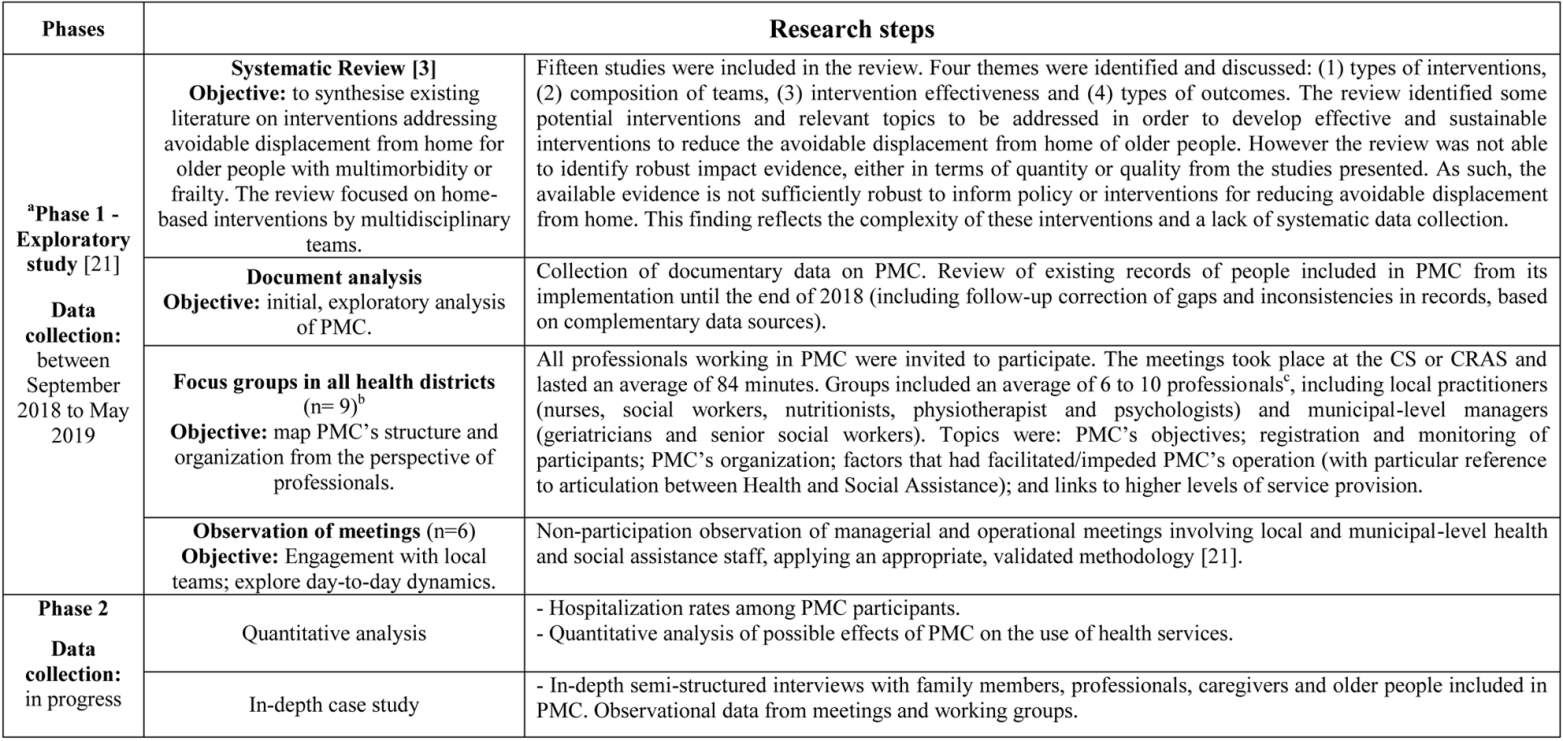
Study design. **a**: In this stage, three Expert Workshop were also held. Objective: networks and dissemination of the first results to public policy makers. **b**: Belo Horizonte, capital of the state of Minas Gerais, is organized into 9 administrative regions (health districts). PMC operates in all administrative regions. **c**: In two focus groups there was also participation of Community Health Agents (*Agente Comunitário de Saúde* – ACS in Portuguese). **Data recording**: Field notes (focus groups and observation of meetings), digitization and filing of the documents collected (document analysis). Organization of a database with the systematization of the main documents and records identified. **Source**: Elaborated by the authors.

The project used a multi-method [22] approach and has two phases (***Figure 1***). This paper draws entirely on Phase 1, as Phase 2 has been delayed as a result of the Covid-19 pandemic. Our data sources include a systematic review of international studies of comparable interventions [3], documentary analysis of PMC’s internal records and new data obtained from focus groups, non-participant observation and expert workshops. Phase 1 focussed on mapping and analysing the development and operation of PMC. Phase 2 (ongoing) focusses on outcomes and impacts.

## Data analysis

The data obtained in Phase 1 were transcribed into Microsoft Word®. We applied three stages of content analysis: pre-analysis (data organization); exploration of the material (definition of analytical categories); and treatment of results, inferences and interpretation (reflective analysis) [23]. This included data coding and thematic analysis and was independently reviewed by two members of the research team in order to reach consensus on the final categories.

Although the specific components of the research design for Phase 1 addressed different objectives, there were important complementarities between them. For example, the document analysis revealed a number of gaps in written guidance about how PMC should operate, including inter-agency collaboration, guidelines and protocols. This in itself is a relevant finding. Consequently, the focus groups served to both fill in some knowledge gaps about how PMC operated in theory, as well as to explore how this compared to daily realities.

## Results

### PMC in theory: objectives and operation

Plans for PMC were first developed in the Department of Health. However, this Department was concerned about the costs of employing paid carers and looked for financial support from the other municipal agency with related interests: Social Assistance. PMC then emerged as a partnership between these two departments, with Social Assistance bearing the full costs of the carers (Document analysis, 2019).

**Figure 2:**
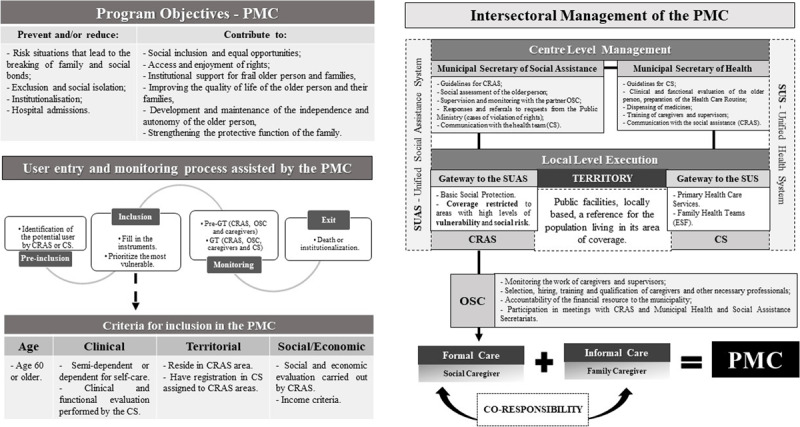
PMC in theory: objectives, criteria and organisational structure. **Abbreviations**: CRAS: Social Assistance Reference Center (*Centro de Referência em Assistência Social* – CRAS in Portuguese); CS: Health Centre (*Centro de Saúde* – CS in Portuguese); GT: Working Group (*Grupo de Trabalho* – GT in Portuguese); Pré-GT: Pre-Working Group (*Pré-Grupo de Trabalho* – Pré-GT in Portuguese); OSC: Civil Society Organisation (*Organização da Sociedade Civil* – OSC in Portuguese); ESF: Family Health Teams (*Equipe de Saúde da Família* – ESF in Portuguese). **Source**: elaborated by the authors, research data. Document analysis, 2019.

The main objectives of the PMC are presented in ***Figure 2***. PMC aims to provide a paid daily carer to semi-dependent and dependent older people identified as clinically and socially vulnerable [18]. The carers only work during normal working hours, but the time spent varies according to the complexity of the case. They are hired through a contract between the municipality and a non-governmental Civil Society Organisation (*Organização da Sociedade Civil* – OSC in Portuguese), responsible for ensuring their training and qualifications, under the supervision of the Departments of Health and Social Assistance. It is also the role of the OSC to select the caregivers (127 in total for the 9 administrative regions), in addition to 02 administrative assistants and 02 general coordinators – who work at the headquarters of the OSC (Document analysis, 2019).

PMC’s management is described as intersectoral, with joint oversight at the central level of the Secretariats of Health and Social Assistance, as well as at the local level through joint activities involving the CS and CRAS teams (***Figure 2***). Each CRAS has staff members with specific responsibility for PMC. At the central level, programme supervisors have overall responsibility for the management of caregivers and for care quality assurance. At the local level, PMC is supported by a close collaboration between families, health professionals, social assistance professionals and PMC carers (Document analysis, 2019).

At the time of Phase 1, PMC covered 62 (40.8%) of the Belo Horizonte’s 152 CS and 26 (76.5%) of its 34 CRAS (its coverage has since increased). However, the geographical territories of CS and CRAS do not always coincide. Some areas covered by a CS do not have a CRAS, and in other areas the same CS corresponds with several CRAS (Document analysis, 2019).

The stages for admission into and monitoring of PMC users are summarised in ***Figure 2***. Pre-inclusion consists of identification of potential users, by a CS or a CRAS or referrals from families, hospitals or neighbours. The criteria for *inclusion* are provided in a set of survey forms which evaluate the older person’s health, functional and social status. After staff from the CS and CRAS complete these forms, applications are prioritised according to the income and social vulnerability of each family, as well as the clinical status and level of functional dependence of the older person. If identified as eligible, the CRAS will meet the family and older person to discuss the scheme and obtain their consent for participation (Document analysis, 2019).

Monitoring activities include a monthly pre-Working Group meeting and the main Working Group meeting. The pre-meeting involves the paid carers, the OSC supervisor and the CRAS team. They agree on practical issues, such as shifts and rotations between caregivers, and discuss the difficulties faced by caregivers related to the care itself or other environmental risks. The main Working Group meeting runs immediately after the pre-meeting, whose participants are joined by health staff (nurses, nutritionists, physiotherapist) and where complex cases of very ill people, problems of familiar violence and discharges from PMC are discussed. Monitoring activities also include monthly and quarterly spreadsheets completed by health staff and sent to the Municipal Office for Health. Older people enrolled in PMC can remain in it for an initial period of up to two years, which can be extended if needed (Document analysis, 2019).

### PMC in practice: preliminary evidence

One of the objectives of the documentary analysis was to collect records for all people included in PMC since its implementation. These include information on age, gender, degree of care dependence and (if leaving PMC) the reason for exiting. This permits some limited descriptive quantitative analysis.

***Table 1*** presents data derived from PMC records for the numbers of older people included in the programme over time, by age and degree of dependence. In each year following 2011 (when PMC began), around a third of people enrolled in PMC (range 29.3% in 2014 to 45.9% in 2018) left the programme and were replaced by new members.

**Table 1:** People included in PMC (by sex and degree of dependence) and rate of renewal (2011–2018)^a^.


YEAR	N	GENDER		DEGREE OF DEPENDENCE		RATE OF RENEWAL

		M^b^	F^c^	D^d^	SD^e^	%

		%	%	%	%	

2011	549	29.51	70.49	45.7	54.3	–

2012	214	28.97	71.03	47.7	52.3	39.00

2013	207	29.47	70.53	48.3	51.7	37.70

2014	161	27.95	72.05	37.3	62.7	29.30

2015	208	32.69	67.31	41.8	58.2	37.90

2016	177	31.07	68.93	39.5	60.5	32.20

2017	212	32.08	67.92	41	59	38.60

2018	252	36.11	63.89	34.9	65.1	45.90

**Average**	**–**	**30.98**	**69.02**	**42.03**	**57.98**	**37.2**


**Note**: a: Total elderly people included in the PMC since its implementation: 1,980 elderly people. **Abbreviations**: b: Male; c: Female; d: Dependent; e: Semi-dependent. **Source**: Elaborated by the authors based on research data. Document analysis, 2019.

As revealed in the documentary analysis, since the implementation of the PMC (in 2011) until the year 2018, 1,980 elderly people were admitted to the Programme. In that same period the average volume of resources invested is around 890,000 dollars a year, which includes the hiring of the caregivers and other professionals necessary for the management of human resources and administrative staff (Field notes – Observation of meetings, 2019).

***Table 2*** presents data for people who left PMC, along with the main reason for leaving. The most common reason was the death of the older person (45.04% of cases). This was unsurprising given the health and functional status of the target population. The second most frequent reason (26.27%) was moving away from the local health district and hence losing local registration with PMC. In 10.94% of cases the main reason given was “institutionalization” (placement into a long-term care facility) and 7.22% of departures from PMC were due to the “rehabilitation” of the older person (so that they no longer needed assistance from PMC).

**Table 2:** Reason for discharge/interruption of participation in PMC by sex and degree of dependence (2011–2018).


REASON	MALE				FEMALE				TOTAL	

	D^a^	%	SD^b^	%	D	%	SD	%	N	%

Change of residence	16	16.33	22	15.07	45	21.74	86	19.72	233	26.27

Family resumes care	21	21.43	22	15.07	56	27.05	97	22.25	196	22.10

Family opt out	24	24.49	26	17.81	49	23.67	69	15.83	168	18.94

Institutionalization	11	11.22	16	10.96	22	10.63	48	11.01	97	10.94

Older person opts out	6	6.12	21	14.38	11	5.31	57	13.07	95	10.71

Risk situation for the caregiver^c^	14	14.29	21	14.38	18	8.70	40	9.17	93	10.48

Rehabilitation of the elderly	5	5.10	17	11.64	6	2.90	36	8.26	64	7.22

No data^d^	1	1.02	1	0.68	–	–	3	0.69	5	0.56

**Subtotal (all reasons above)**	98	36.98	146	60.58	207	45.59	436	66.67	887	54.96

**Death**	167	63.02	95	39.42	247	54.41	218	33.33	727	45.04

**Total**	265	100.0	241	100.0	454	100.0	654	100.0	1614	100.0


^a^: Dependent; ^b^: Semi-dependent; ^c^: Lack of security in the territory of operation; ^d^: No reason provided. **Source**: Elaborated by the authors based on research data. Document analysis, 2019.

**Figure 3:**
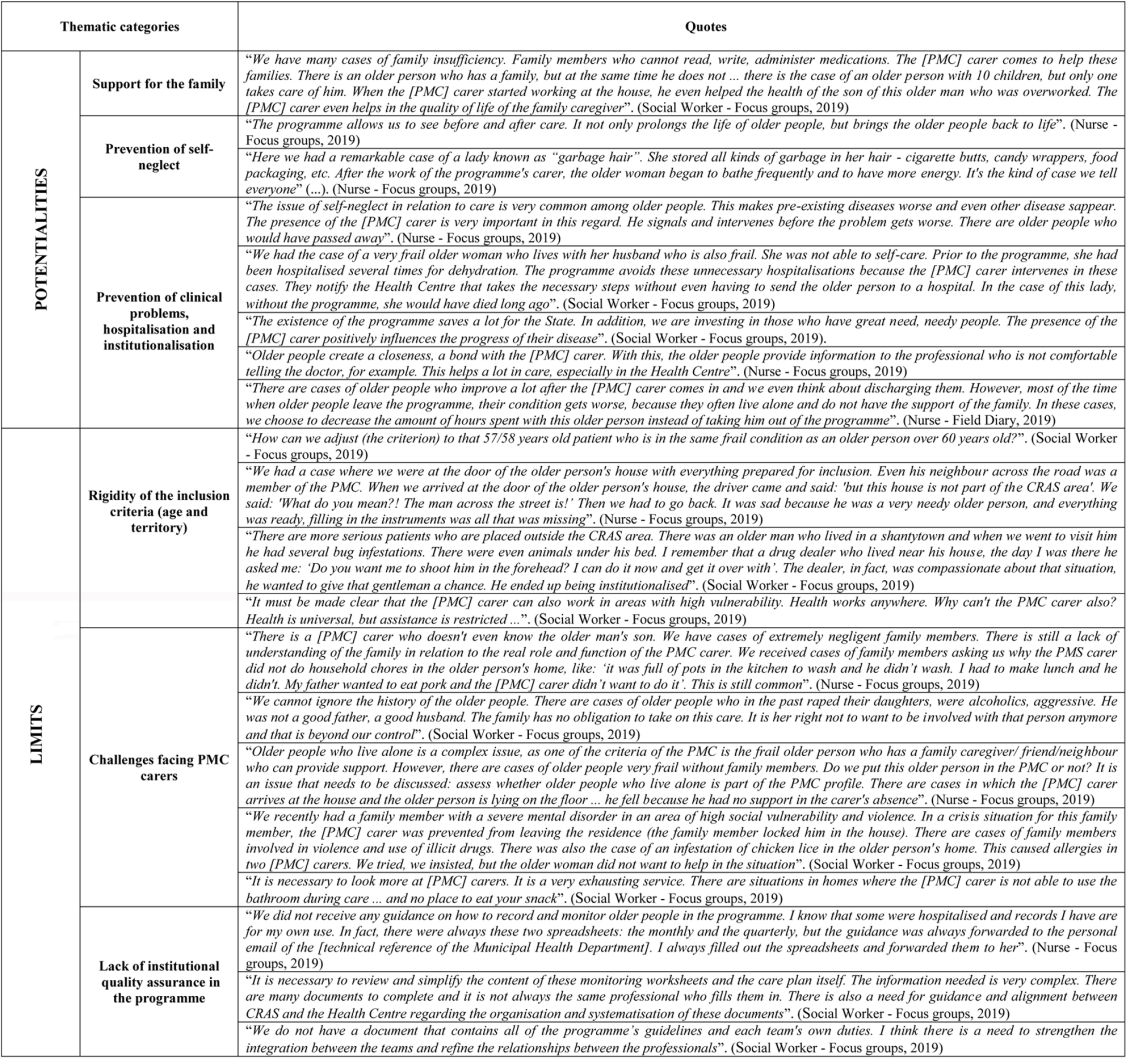
Potential and limits of the PMC – professionals’ perspective. **Source**: Testimonials collected during the meetings focus groups. Field notes, 2019.

During the focus groups professionals affirmed that PMC appears to be appreciated and valued by all those involved, namely the users, professionals and family members. It was possible to identify three main thematic categories that highlighted the potential of the programme, namely: *support for the family; prevention of self-neglect and prevention of clinical problems, hospitalisations and institutionalisation*. ***Figure 3*** provides quotes from participants and recorded observations to illustrate these thematic categories. Although there was a strong consensus from study participants that PMC “mainly works well” on a day-to-day basis, a number of issues and frustrations were also identified. These were framed in terms of areas for improvement, rather than grounds for discontinuing PMC. These concerns are summarised in ***Figure 3***.

Staff questioned the *Rigidity of the inclusion criteria – age and territory* (***Figure 3***). Potential inclusion in PMC was restricted to people aged 60 or more living in defined administrative zones served by specific CRASs. This could potentially exclude some vulnerable families and some CSs staff referred to the frustration of identifying older people as potentially eligible for inclusion in PMC, only to discover that they did not live within a neighbourhood linked to a CRAS. Staff recognised that these rigid criteria were mainly applied to manage high levels of demand for inclusion in PMC, which greatly exceeded their resourcing capacity. They saw this as part of a wider problem of under-resourced community services, observing that both the CSs and CRASs are small, understaffed, poorly maintained and unable to meet the needs of their communities (Field notes – Focus groups, 2019). As such, rather than an inherent criticism of PMC itself, these concerns reflected a wider frustration with the weakly resourced setting within which it operated.

A separate set of concerns related to the many challenges facing PMC carers who were working with vulnerable families in highly deprived neighbourhoods – *Challenges facing PMC carers* (***Figure 3***). This could expose PMC carers to threatening situations and generate a range of tensions and misunderstandings. These included challenges in establishing which family members had the main care responsibility for the older person and in distinguishing between the roles of PMC carers and those of health care professionals. There were sometimes tensions between the theoretical premise that families would cooperate closely with PMC carers and the complexities of dealing with highly vulnerable individuals. The capacity of family members to work alongside PMC carers to support older relatives was sometimes limited due to overload, a lack of education and problematic personal relations between relatives (Field notes – Focus groups, 2019). Again, rather than an inherent weakness of PMC, these concerns reflected the hugely challenging contexts in which it operated.

A third set of concerns were more integral to PMC itself – *Lack of institutional quality assurance in the programme* (***Figure 3***). These refer to the limited institutional embeddedness of the programme and challenges in cross-agency collaboration. These concerns were supported by our documentary review which found few official materials defining PMC’s formal institutional status, management guidelines or collaboration protocols. Instead, there was evidence that relationships between different agencies and ways of working together, both at the community and municipal levels, had been allowed to develop organically as the programme evolved. This may have permitted some flexibility through common sense learning-by-doing, but had impeded the formalisation of these arrangements. The absence of comprehensive written protocols occurred in a context of high staff turnover at both the municipal and community levels, generating confusion about roles and responsibilities (Field notes – Focus groups, 2019). According to some focus group participants, CS health teams did not always meet PMC expectations due to the many demands on their time and a lack of specific protocols.

A direct consequence of the limited formalisation of PMC was inconsistent record keeping and a lack of information sharing across different agencies (Field notes – Focus groups, 2019). The application of monitoring instruments (monthly and quarterly spreadsheets) was inconsistent across different areas. There were gaps in the PMC data systems operated by both the departments of Health and Social Assistance and they were mutually incompatible, thus preventing data sharing (Field notes – Focus groups and Document analysis, 2019). Separately from this, PMC carers were required to write up daily case notes and compile these into monthly reports for the Civil Society Organisation which had originally recruited and trained them. These records were not shared with the rest of the Health and Social Assistance teams and most professionals involved in PMC did not understand the purpose of this exercise or the meaning of the data (Field notes – Focus groups, 2019).

**Figure 4:**
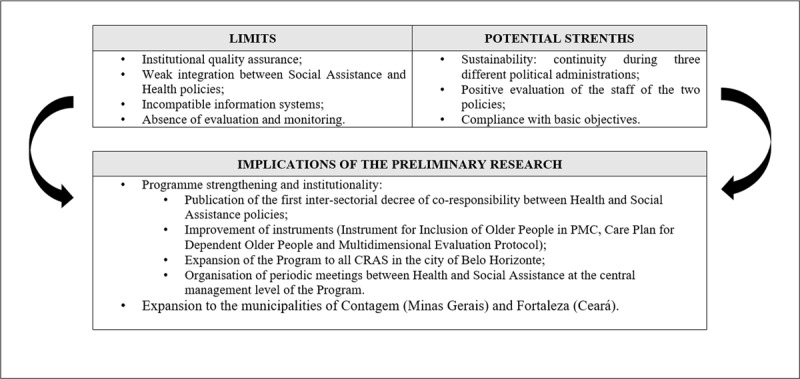
Limits and potential strengths of PMC. **Abbreviations**: CRAS: Social Assistance Reference Center (*Centro de Referência em Assistência Social* – CRAS in Portuguese). **Source**: elaborated by the authors based Observation of meetings. Field notes, 2019.

In sum, our data revealed that, although PMC was almost universally viewed as highly effective, the programme was not without flaws and areas of weakness. These strengths and limitations, summarised in ***Figure 4***, mainly reflected PMC’s somewhat *ad hoc* evolution and the challenges of working with vulnerable families. Having operated along somewhat informal lines for several years, the view of most study participants was that the time had come to take PMC “to the next level”, with clearer and more consistent understandings. Following the presentation of these findings to the Departments of Health and Social Assistance, steps have been taken to new develop simpler and more systematic records, as well as enhanced protocols for data sharing across agencies. A new official document sets out the main elements of PMC, as well as the specific roles and responsibilities of different agencies [24]. This is the first document to set out this information and, as such, represents a key step towards formalising PMC’s institutional status. More generally, the evident strengths of PMC revealed in our study led to the expansion of the programme to all previously unserved CRAS in Belo Horizonte and the piloting of similar schemes in other cities.

## Discussion

This study presents results from the first phase of evaluation of an innovative integrated health and social care programme for older people in Brazil. These data must be analysed with reference to the wider national context. Although Brazil has an extensive legal framework for supporting older people, public policies are still not being fully implemented. Brazil has mainly focussed on providing contributory pensions and means-tested cash benefits for older people [25]. However, these income transfers are not enough to address the care needs of a population that is ageing in difficult social and economic conditions. There is little coordination between federal departments responsible for health and social care. This disconnect feeds down to lower tiers of government, including at the district level where separate infrastructures are based around CS and CRAS whose territories in some cases do not coincide.

It should be noted that the Program should serve a much larger number of people when compared to the total elderly population in the region studied. Currently the city of Belo Horizonte has about 380,000 elderly people, of whom it is estimated that 8% would be incapacitated in basic activities of daily living [26] and 75% totally dependent on the Unified Health System.

When it comes to comparing the theoretical aims, objectives and ambitions of PMC with the practicalities of implementation, there are a number of contrasting issues. Just as in other countries, the experience of PMC demonstrates that effective intersectoral cooperation is a major challenge, especially in contexts of high clinical and social vulnerability. Notwithstanding positive statements by stakeholders on the scheme, the problems identified were similar to other studies of integrated health and social care interventions in high-income countries and referred to difficulties due to the complexities of existing professional communication pathways and incompatibility of systems [27,28]. The inclusion of other professionals such as mental health teams and community doctors is also seen as desirable, but often unfeasible due to competing priorities and resource deficits [29]. Some barriers to effective collaboration are more specific to counties like Brazil. For example, Brazil has very high rates of staff turnover in health and welfare agencies, among other things, due to temporary contracting and a tendency for new governments to replace staff with their “own people” [30].

There are clear discrepancies between theoretical expectations about collaboration with families (which to some degree reflected Latin American cultural norms and ideals of family care-giving) and the challenging realities of struggling families in highly-deprived communities [31]. Laws requiring families to care for older people need to consider their real capacities to do so [32]. By offering family carers respite and support, PMC goes some way towards recognising these challenges, but is not always able to resolve them. Nevertheless, PMC shows the value of interventions that see family support, along with formal health and social care provision, as complementary parts of an integrated support system for frail older people [17].

Despite the challenges outlined, PMC represents a pioneering programme that has been able to establish a meaningful partnership between local departments of health and social assistance and to sustain this partnership over nearly a decade. These positive perceptions contributed to decisions to sustain PMC over two different municipal administration. This is unusual in Brazil, where programmes of this nature rarely outlast a single electoral cycle [33]. They also contributed to a decision, in 2019, to extend PMC into new neighbourhoods, to strengthen its information systems and to formalise its institutional design. These developments reflect the appetite of policymakers in Brazil and other middle-income counties to identify more effective approaches to meet the health and social care needs of their fast-growing older populations [34,35]. PMC can also be understood as a social innovation, promoting Ageing in Place through adequate health and social support [17,36,37].

## Limitations and strengths of the study

These results analyse the structure and operation of PMC, but do not explicitly discuss its effects or impacts. Clearly, the two are linked, inasmuch as effective operation and intersectoral coordination are conducive to more positive impacts, and general comments about PMC from a range of participants and stakeholders were favourable.

A strength of our research design is its use of multiple methods: document review, focus group discussions and non-participant observation. This permitted an element of validation and consistency checking across data, although some of the qualitative data may reflect the subjective biases and preferences of participants [38,39]. The second phase of the study will develop a more substantial set of data from different sources, including the perspectives of older people and families participating in PMC.

## Conclusion

The insufficient provision of adequate services facing the new demographic condition affects the daily routine of users, the community and the Health and Social Assistance systems. Intersectoriality is another challenge, especially in contexts of high clinical and social vulnerability.

The Older Adult Care Programme is a beacon of integration between the two social protection public policies in the Brazil. This research allowed to identify the potential of the Programme, promoting its strengthening and institutionalization. Notwithstanding the challenges PMC has faced and its sometimes *ad hoc* operation, overall comments about the programme from a range of participants and stakeholders were strongly favourable.

However, some gaps were identified in the implementation of the Program, which corroborates the need to systematically monitor its execution, not to point out errors and flaws, but to improve processes that guarantee its effectiveness. The preliminary findings from this study also provided a clearer description of the challenges and considered the extent to which they reflect specific problems with the PMC itself or are the result of other bigger issues, such as a lack of resources.

Although the results of this study are limited to a level of local action, it is observed that supplement the complex health needs with limited resources is a global challenge.
